# Pediatric HIV Long-Term Nonprogressors

**DOI:** 10.1155/2014/752312

**Published:** 2014-08-26

**Authors:** B. H. Rimawi, R. H. Rimawi, M. Micallef, L. Pinckney, S. L. Fowler, T. C. Dixon

**Affiliations:** ^1^Department of Obstetrics and Gynecology, Division of Reproductive Infectious Diseases, Division of Maternal Fetal Medicine, Emory University, 550 Peachtree Street NE, Atlanta, GA 30308, USA; ^2^Division of Infectious Diseases and Critical Care, Department of Internal Medicine, Emory University, 550 Peachtree Street NE, Atlanta, GA 30308, USA; ^3^Division of Neonatology, Department of Pediatrics, Medical University of South Carolina, 96 Jonathan Lucas Street, Charleston, SC 29425, USA; ^4^Department of Obstetrics and Gynecology, Medical University of South Carolina, 96 Jonathan Lucas Street, Charleston, SC 29425, USA; ^5^Division of Infectious Diseases, Department of Pediatrics, Medical University of South Carolina, 96 Jonathan Lucas Street, Charleston, SC 29425, USA

## Abstract

Patients infected with HIV are best categorized along a continuum from rapid progressors to HIV long-term nonprogressors. Long-term nonprogressors (LTNPs) are those in which AIDS develop many years after being infected with HIV, often beyond the 10-year mark, and represent 15–20% of the HIV infected patients. Many of these patients are able to control their infection and maintain undetectable viral loads for long periods of time without antiretroviral therapy. After a comprehensive literature search, we found extensive data related to HIV LTNPs in the adult population; however, very limited data was available related to LTNPs within the pediatric population. We present a case of pediatric HIV LTNPs, perinatally infected patient with undetectable viral loads, despite never receiving ART. Although there are not many instances of LTNPs among children, this child may be one, though she had intermittent viremia. She has continued to manifest serologic evidence of infection, with yearly ELISA and western blot positive tests. Based on the viral fitness studies that were performed, this case exemplifies an adolescent LTNP.

## 1. Introduction

When patients are infected with HIV, their disease process can be best characterized along a continuum from rapid progressors to HIV long-term nonprogressors (LTNPs). Subsets of these patients are characterized as rapid progressors, in which they develop AIDS within a few years, often less than 5 years. In contrast, long-term nonprogressors are those in which AIDS develop many years after being infected with HIV, often beyond the 10-year mark, and represent 15–20% of the HIV infected patients. LTNPs are a subset of long-term nonprogressors, generally referred to as individuals who have contracted the HIV infection but control their infection and maintain undetectable viral loads for long periods of time without antiretroviral therapy [1].

During our literature search, we found extensive data related to HIV LTNPs in the adult population; however, very limited data was available related to LTNPs within the pediatric population. It is important not to confuse pediatric LTNPs with pediatric slow progressors, as the latter is referred to a group of HIV-1-infected pediatric individuals who are homogeneous for route and length of infection and standard of care, thereby having disease progression requiring antiretroviral therapy at some point of their lives. We present a case of pediatric HIV LTNPs, perinatally infected patient with undetectable viral loads, despite never receiving ART.

## 2. Case Report

A 14-year-old female with a history of HIV acquired via vertical transmission from a noncompliant, advanced AIDS-infected mother presented to our tertiary care hospital. She had no siblings and lived with her mother for the first two years after birth until her mother died of an AIDS-associated infection. Information regarding the HIV status of her biological father was unknown, as this individual was never involved in her care. She missed all her medical appointments while living with her mother. Afterwards, she resided with her maternal grandparents. At that time, she had her first medical evaluation without prior antepartum medical records, where an ELISA and western-blot test were done and came back positive. Her HIV-1 plasma viral load was 1,050-copies/mL and a CD4 count was 2,869 cells per cubic millimeter of blood (CUMM). HIV-1/2 antibody testing confirmed an HIV-1 infection.

She was transferred to an HIV satellite clinic and later to our tertiary infectious disease adolescent clinic. She had never taken ART prior to her MUSC visit. Tables 1 and 2 demonstrate the values of her immunodeficiency panel as well as her HIV-1 viral load since initiation of care at our center. Her repeat HIV-1 plasma RNA viral load was 632-copies/mL with a CD4 count of 2,364/cumm. She was monitored clinically without ART and serial repeat laboratory tests were performed.

Her HIV-1 plasma viral load remained undetectable and her viral load remained dramatically unchanged. Repeat ELISA and western blot testing performed on a yearly basis since initial HIV diagnosis have been positive (Table 2). Of note, further investigation was significant for a positive human leukocyte antigen B27 test; however, no deletions or mutations were appreciated. Additional testings for ankylosing spondylitis, reactive arthritis, sacroiliitis, uveitis, and other conditions known to have a positive HLA-B27 antigen test were negative. Proviral HIV tropism testing confirmed a CCR5-tropic (R5) using HIV-1 virus.

Two years later, her viral load rose to 614-copies/mL of plasma. Her viral load remained between undetectable and 727-copies/mL and her CD4 ranged from 1151 to 2186/cumm despite not being initiated on ART. Since 2005, it has remained undetectable without ART. With respect to her overall general clinical course, she has not had any complicating opportunistic infections, hospitalizations, or complications related to HIV. She is a hitherto fit girl, with all the appropriate signs of puberty and sexual development for her age group, including her height and weight.

## 3. Discussion

Pediatric long-term nonprogressors (LTNPs) are individuals infected with HIV-1 who maintain a suppressed viral load, which refers to either an undetectable viral load (HIV RNA <20 to 75 copies/mL) or a low-detectable HIV-RNA viral load (typically HIV RNA <200 copies/mL), depending on the sensitivity of the HIV RNA assay that is used, despite not being on ART [2]. The clinical relevance of the LTNP classification is not well understood, as some patients remain virologically suppressed, while others have gone on to develop AIDS. LTNPs generally remain at a certain viral load despite not receiving ART [1]. Unlike patients with a “functional” cure, these patients are once known to have detectable HIV-1 RNA viral loads, similar to our index case of ~20,000 copies/mL of plasma, and have spontaneously suppressed their viral load and maintained a stable CD4 count without ART influence. Of importance, in comparison to LTNPs, patients with a “functional” cure would test negative for HIV at some point of their care, whereas LTNPs will always have a positive HIV test [2].

After acquisition of HIV, the virus naturally enters into human cells and attach to proteins on that cell. HIV tropism testing allows for knowledge to the type of protein that HIV attaches to. These proteins are the CCR5 and CXCR4, and most HIV infections are caused by the CCR5-using virus; however, as HIV reproduces, it may change from a CCR5-using virus to a CXCR4-using virus. Mutations or deletions in these proteins can account for why an HIV-infected individual rarely progresses to AIDS. Our index patient had a CCR5-tropic using HIV-1 virus.

Different etiologies have been studied which allows long-term nonprogressors to go live without taking ART. These etiologies consist of gene and receptor mutations [3], various mitochondrial DNA types [4], and different human leukocyte antigen (HLA) types, specifically HLA-B27 or HLA-B57 [5]. Perinatally infected children with HIV may acquire the same genes and HLA as their mothers. However, since differences in transcriptional regulation have been observed among different HIV-1 subtypes, the viruses may also be very different. With our index patient, whose mother died secondary to opportunistic infections complicating her advanced AIDS, HLA antigen testing was positive for HLA-B27; however, no deletion or mutation was appreciated.

One question that arises is, was her biological father also HIV-infected? And if so, the possibility of this patient acquiring a mutated HLA B27 gene must be entertained. Unfortunately, her biological father was not involved in her care and his exact presence was unknown; therefore, testing for this was not available and therefore would remain a mystery. Although there are not many instances of LTNPs among children, this child may be one, though she had intermittent viremia. She has continued to manifest serologic evidence of infection, with yearly ELISA and western blot positive tests. Based on the viral fitness studies that were performed, this case exemplifies an adolescent LTNP.

## Figures and Tables

**Table 1:** Clinical laboratory results: CD4/CD8 values.

Date	CD3%	^€^CD3 absolute #	CD4%	^€^CD4 absolute #	CD8%	^€^CD8 absolute #	CD4/CD8 ratio
	(*n*: 52–78)	(*n*: 800–3500)	(*n*: 25–48)	(*n*: 400–1200)	(*n*: 9–35)	(*n*: 200–900)	(*n*: 0.9–3.4)
05/07/2009	72	2106	40	1151	29	842	1.4
01/08/2009	74	2049	44	1229	26	713	1.7
08/28/2008	74	1967	43	1152	26	699	1.7
05/29/2008	75	1736	50	1149	22	510	2.3
03/09/2006	73	2240	47	1431	21	649	2.2
11/10/2005	72	2233	44	1359	22	689	2.0
07/21/2005	72	2562	43	1482	26	903	1.6
12/02/2004	74	2806	42	1681	23	928	1.8
03/11/2004	71	3370	51	2186	21	912	2.4
04/14/2003	80	3194	43	1890	23	1041	1.8
12/09/2002	72	3779	43	2153	26	1310	1.6
08/19/2002	75	3976	44	2338	25	1327	1.8
04/15/2002	74	3798	39	2120	24	1328	1.6
01/07/2002	72	3989	45	2517	21	1189	2.1
11/01/2001	62	3699	31	1852	25	1496	1.2
08/09/2001	68	5386	37	2971	23	1868	1.6
05/23/2001	69	4821	31	2686	25	1752	1.3
02/12/2001	70	3706	44	2364	20	1076	2.2

^€^mm^3^: cells per cubic millimeter.

*n*: normal reference interval for patient's age (Medical University of South Carolina).

**Table 2:** Clinical laboratory results: HIV viral load.

Date	HIV-1 plasma viral load	ELISA/western blot testing
	(*n*: <40 copies)	(*n*: negative)
05/07/2009	UD^¥^	Positive
01/08/2009	UD	Not performed
08/28/2008	UD	Positive
05/29/2007	UD	Positive
03/09/2006	UD	Positive
11/10/2005	UD	Positive
07/21/2005	726	Not performed
12/02/2004	320	Not performed
03/11/2004	476	Positive
04/14/2003	614	Positive
12/09/2002	UD	Not performed
08/19/2002	UD	Not performed
04/15/2002	UD	Not performed
01/07/2002	UD	Positive
10/01/2001	UD	Not performed
07/09/2001	UD	Not performed
04/23/2001	UD	Not performed
02/12/2001	632	Positive

^¥^UD: undetectable HIV-1 plasma viral load/copies per milliliter.

*n*: normal reference interval (Medical University of South Carolina).

Threshold for detection is any HIV-1 viral load >40 copies/mL.

Method used to quantify HIV-1 plasma RNA viral load: real time PCR.
